# Sacral neuromodulation - when and for who

**DOI:** 10.1590/S1677-5538.IBJU.2021.99.08

**Published:** 2021-03-25

**Authors:** Marcelo Mass-Lindenbaum, D. Calderón-Pollak, H. B. Goldman, Javier Pizarro-Berdichevsky

**Affiliations:** 1 Universidad de los Andes Santiago Chile Universidad de los Andes, Santiago, Chile.; 2 Glickman Urologic Institute Cleveland Clinic ClevelandOH USA Glickman Urologic Institute, Cleveland Clinic, Cleveland, OH, USA.; 3 Hospital Sótero del Río Centro de Innovación en Piso Pélvico Santiago Chile Centro de Innovación en Piso Pélvico, Hospital Sótero del Río, Santiago, Chile.; 4 Universidad Católica de Chile Hospital Sótero del Río Division de Obstetricia y Ginecología Santiago Chile Division de Obstetricia y Ginecología, Hospital Sótero del Río, Pontificia Universidad Católica de Chile, Santiago, Chile.

## WHAT IS SACRAL NEUROMODULATION?

Sacral neuromodulation (SNM) is a therapeutic technique that involves electrical stimulation to a sacral nerve root to modulate a neural pathway. It is a minimally invasive procedure with good long-term outcomes used to treat bowel and/or bladder dysfunction (1).

SNM is currently offered to patients with idiopathic overactive bladder (OAB) syndrome with or without incontinence, fecal incontinence (FI) and non-obstructive urinary retention (NOR), who have failed to respond to conservative or medical therapies, such as pelvic-floor physical therapy and behavioral changes, that are the treatments typically considered as the first approach (1).

It can also be used as a therapeutic option in both bladder pain syndrome and neurogenic lower urinary tract dysfunction (NLUTD) who are at low risk of upper urinary tract deterioration, but the evidence is still very limited (level III/ grade of recommendation C) (1).

## HOW IT IS THOUGHT TO WORK

Normal urinary function is controlled by brain and other peripheral neurologic structures (2, 3). Recent studies with functional magnetic resonance imaging (fMRI) have shown that certain brain areas, considered to be associated with urinary symptoms and urge, are hyperactivated in patients with OAB (4–6). Adding to this, it was seen that brain activity in female patients with OAB who had received pelvic floor physiotherapy (PFPT), with successful results, differed from those women to whom PFPT was ineffective in fMRI (7).

It is thought that SNM modulates neural circuits in both central and peripheral pathways, though its mechanism is not completely elucidated. This was made evident after fMRI revealed real-time changes in brain activity of OAB patients who had received SNM, supporting the idea that SNM functions in part, through regulations in some pathways of the central nervous system (7). Additionally, with altered stimulus intensity, an amplitude-response association was found in various cerebral locations. What still remains unclear is how normalization of the lower urinary tract function relates to brain activity changes with SNM (7).

There are currently two hypotheses to understand SNM's therapeutic mechanism (7).

The first one, suggests that SNM produces afferent activity that overrides or interferes with the aberrant neural activity underlying OAB, similar to how noise-cancelling headphones can allow soft music to be heard in a noisy environment (7).

A second theory states that it produces neuro-modulation, which could alter the set-point for voiding in brain centers that coordinate lower urinary tract function, similar to how a thermostat can be adjusted to maintain a comfortable temperature. In OAB, this set-point is likely miscalibrated, underlying the disease state (7).

## CURRENT INDICATIONS

SNM indications for OAB are as follows per American Urologic Association (AUA) and International Continence Society (ICS) guidelines:

According to AUA “Clinicians may offer sacral neuromodulation as third-line treatment in a carefully selected patient population characterized by severe refractory OAB symptoms or patients who are not candidates for second-line therapy and are willing to undergo a surgical procedure. Recommendation (Evidence Strength Grade C)” (8).

According to ICS “SNM can be offered to patients with OAB with or without incontinence who fail to respond to or are intolerant of conservative and medical therapies. (Level of Evidence: I; Grade of Recommendation: A)” (1).

It is very important to note that OAB can present itself with or without incontinence. This can lead to a classification of OAB into “dry” and “wet”.

In the case of OAB without incontinence or “dry”, the ICS quotes a randomized, prospective, 12 center study which enrolled 51 patients who had OAB “dry” or a severe urgency-frequency syndrome. In this study, patients who showed a satisfactory response to peripheral nerve evaluation (PNE), which is a trial with a temporary sacral lead, where assigned randomly to immediate treatment or to the control group that consisted of implant after a delay of 6 months.

After 6 months, voiding diary results showed improvements that were statistically significant in the immediate implant group, as to degree of urgency, number of daily voids and volume per void, in comparison to the control group (9).

In another study that had 2 year follow up, 29 patients with urgency- frequency syndrome (or OAB “dry”) showed a significant reduction in the total of voids per day, with 56% of them showing a reduction of 50% or more in their average voids per day (10).

In the case of OAB with incontinence or OAB “wet” SNM also showed positive results at medium and long terms.

A prospective, multicenter, randomized trial included 34 patients with severe OAB “wet” (urge-incontinence) who had an immediate implant after a positive trial test; and 42 patients as controls who received only standard medical therapy for 6 months and then had the device implanted (delayed group).

After 6 months, the immediate implant group showed a significant reduction in number of UI episodes, severity, and number of daily diapers or absorbent pads replaced, compared to the control group.

In the immediate implant group, 16 (47%) patients were completely dry and 10 (29%) other patients showed a >50% reduction in UI episodes at 6 months of follow-up (11). After 18 months, the efficacy of the treatment remained high and surgical revision was required in 32.5% of the patients.

Around 10% of the patients had to have a device explant because of lack of efficacy, bowel dysfunction or pain. No permanent therapy-related injuries were reported (11).

After 3 years, symptoms like leaking were significantly reduced with 59% of the patients reporting a ≥50% reduction in leaks per day and 46% of the patients reported being completely dry (10).

A study with a median follow-up of 50.7 months reported a success rate of 84.8% for UUI. In this study39% of patients needed revision of the SNM neuromodulation implant (9).

At 6 months, SNM showed superior objective and subjective results compared to standard pharmacologic treatment for OAB.

SNM is shown to be a safe and effective treatment for OAB patients (10).

In 2009, a Cochrane review concluded that implantable neurostimulators have benefits for some patients with OAB symptoms, retention without organic obstruction, and in those for whom other methods of treatment have failed (12).

In 2016, the ROSETTA trial, a multicenter randomized trial with 381 patients, that compared SNM with Onabotulinumtoxin A as a treatment for refractory UUI, showed a slightly greater improvement of daily UUI episodes in the Onabotulinumtoxin A compared to the SNM group, but the need for intermittent catheterization and UTI episodes were more frequent in the Onabotulinumtoxin A group (13). However, this study has been criticized by many as the dose of Onabotulinumtoxin A utilized was twice the standard approved dose and the SNM lead that was utilized is an older one that is no longer used. This may account for some of the improved efficacy of Onabotulinumtoxin A.

A more recent trial, the ARTISAN trial, with 129 patients, showed that 90% of the patients had a ≥50% reduction of daily UUI episodesat 6 months follow up.

In the cited trial, the mean UUI episodes were reduced from approximately 5.6 episodes per day, to approximately 1.3 (14).

Considering all of the above, SNM is shown to be a safe and effective treatment for OAB patients (10).

### SNM indications for Idiopathic non-obstructive retention (NOR) are as follows per ICS guidelines:

“SNM is an effective treatment for Fowler's Syndrome, voiding dysfunction and NOR. (Level of Evidence: I; Grade of Recommendation: A)” (1);

“In general patients with NOR, usually confirmed with urodynamics, are offered to use intermittent self-clean catheterization. However, some patients are not able to do so or are not satisfied with this option. In these patients SNM should be considered as an option” (1).

A prospective, randomized study that included 12 centers and 177 patients with NOR, demonstrated a 71% clinical success rate after 5 years of implantation of the SNM. All patients had a PNE, and 38.4% received the implant. Of the 68 patients who qualified for implantation, 37 were randomly assigned to an immediate treatment and 31 to a 6-month delayed implant (control group). After 18 months, 70% of the patients who had received the implant (immediate or late), showed more than 50% reduction in volume per catheterization. Further publications showed that 69% of the patients treated with implants eliminated catheterization at 6 months and an additional 14% had more than a 50% reduction in volume per catheterization. After 6 months, successful results were achieved in 83% of the implant group with NOR in comparison to 9% of the control group.

Adding to this, a short-term inactivation of SNM showed a significant increase in residual volumes (15). A 5-year follow-up evidenced an important reduction in the mean volume per catheterization and of the mean number of catheterizations (15, 16).

A single center study showed a spontaneous voiding rate of 72% out of 60 patients implanted, over a follow-up of 4 years. After the procedure, of the 43 women who voided, only 13 required the continued use of intermittent self-catheterization twice a day, but this still was less than before the surgery. Patients who had abnormal sphincteric EMG before surgery had better results, with 76% of them experiencing complete restoration of voiding (17). Another study concluded that Fowler's syndrome is a positive predictive factor for SNM in female urinary retention (18).

Various single-center studies reported satisfactory long-term results ranging from 73% (19) to 83% (20).

### SNM indications for fecal incontinence (FI) are as follows per ICS guidelines and American Society of Colon and Rectal Surgeons’ (ASCRS) clinical practical guideline for the treatment of fecal incontinence:

- According to the ICS guidelines: SNM should be considered as a second line treatment option for bothersome FI in patients who have failed conservative measures. (Level of Evidence: 2, Grade of Recommendation: B) (1);
- According to the American society of Colon and Rectal Surgeons, “Sacral neuromodulation may be considered as a first line surgical option for incontinent patients with and without sphincter defects. (Grade of Recommendation: Strong recommendation based on moderate-quality evidence, 1B) (21).

Both the ICS and the ASCRS consider SNM to be a first line surgical treatment for FI, even though the ICS considers SNM as a second line treatment, this is only because they consider conservative measures as a first line of treatment, but when considering a surgical option SNM is the first option.

The ICS and ASCRS both base their statements on the same evidence, that indicates that SNM has shown to reduce the frequency of FI episodes (22–26). They both quote a systematic review that showed that approximately 79% (69%-83%) of patients experience ≥50% improvement in weekly FI episodes at 12 months follow up and 84% of patients experience ≥ 50% improvement at > 36 months follow-up. It is worth noting that this review was only in patients who received a full system implant (26).

They both agree that the presence of a sphincter injury does not appear to impact the outcome of SNM (21). This is based on various studies that demonstrate that either an external or internal sphincter defect is not a formal contraindication for SNM (27–37).

The ICS goes further, by also stating that the size of the muscle defect does not affect decision making. This is because SNM relies more on sensory fiber stimulation than a motor response (1, 38, 39).

Brouwer et al. (34) demonstrated that the presence of a pudendal neuropathy, a sphincter defect, or a previous sphincter repair did not affect the efficacy of SNM.

Even though there is excellent evidence proving long-term success, there is only one study that compares it to another surgical technique. This study shows a comparison between 15 patients with an SNM device and 15 control patients with an artificial bowel sphincter (ABS). Even though postoperative incontinence scores (Cleveland Clinic Florida fecal incontinence or CCF score) were moderately better in the (ABS) group, postoperative constipation scores were slightly worse and postoperative quality of life did not differ (40).

The ICS mentions a special group of patients that could benefit from SNM and these are patients with FI after a Low Anterior Resection for rectal cancer (1).

As time passes, treatment for rectal cancer improves and sphincter preservation strategies have been optimized. Many of these patients are cured of their cancer, but around 50-90% will suffer bowel dysfunction and many of them will suffer low anterior resection syndrome (LARS), which includes FI, fecal urgency and frequent bowel movements, which could be very bothersome or even debilitating. (41–44) Because these patients have an anatomical alteration, it is still unclear how much benefit they may have with SNM in achieving symptomatic relief.

Two studies were conducted to establish whether SNM was a good option in patients with LARS. 47-100% of patients who had a basic SNM peripheral nerve evaluation (PNE) had success in symptomatic amelioration (42), but it has to be noted that the groups of patients were heterogeneous, so the information was difficult to analyze. Also, LARS is a syndrome that consists of different symptoms such as bowel movement clustering, urgency, and FI. Though most studies report an improvement in continence, future studies should include a more integral scoring system, like the LARS score (45), to understand which elements of the overall syndrome are improved by SNM.

The current recommendation by the ICS is that SNM should be considered after lifestyle modifications and medical treatment. (Level of Evidence: 3, Grade of Recommendation: D) (1).

Another interesting scenario where the ICS proposes SNM as a treatment option is in patients with combined bowel and urinary symptoms.

Two studies showed encouraging results regarding SNM in patients with mixed symptoms. One study of 14 patients showed excellent results in these patients who had FI associated with urinary symptoms (46). The second study, analyzed 24 patients who had both UI and FI, and showed symptomatic improvement in more than 30% who used SNM with a mean follow up of 28 months. With this information, SNM could be helpful in a selected group of patients who have a combination of UI and FI, as it has showed significant improvement in both urinary and bowel symptom scores (47, 48).

Additionally, SNM has been studied in pediatric patients who have a combination of UI and FI and showed good results. Prospective clinical data and patient-reported measures, 29 patients, show an improvement that ranges between 55-91% (48).

Even though the level of evidence is low (Level of Evidence: III, Grade of Recommendation: C) SNM is the preferred therapy for these patients (1).

### SNM indications for Interstitial Cystitis (IC) /Bladder Pain Syndrome (BPS) as per ICS guidelines are as follows

IC/BPS is a condition characterized by urinary frequency, urgency, nocturia, but most importantly, bladder, urethral and pelvic pain (48). There is limited evidence supporting the use of SNM in patients with IC/BPS. Typically these are small observational case studies with different criteria for success. Nevertheless, SNM may be considered in patients with IC/BPS who do not sufficiently respond to first, second or third-line treatments. Based on these small observational studies, the success rate for SNM for IC/BPS was 48% to 72% (50–55).

Though SNM may be considered in IC/BPS as a fourth-line of treatment option there is minimal evidence reporting its efficacy in non IC/BPS chronic pelvic pain (56). Based on available studies, SNM cannot be recommended as a treatment option in these patients. However, pelvic pain is not a contraindication in patients with concomitant voiding symptoms such as urgency and frequency.

## POSSIBLE FUTURE INDICATIONS

### Constipation:

A Cochrane systematic review done in 2015 showed a limited number of studies in this population and reported no important improvement of fecal frequency in the only crossover trial that compared SNM and sham stimulation in 59 patients with refractory constipation (57).

In a systematic review including 7 studies regarding SNM use for constipation and FI in a pediatric population, all studies indicated a significant improvement in constipation that was refractory to conservative medical treatment, with persistent enhancement in the following two years. Nevertheless, complication rates fluctuate between 17 and 50% in all studies (58).

### Neurogenic bladder:

An important barrier for the use of SNM in patients with neurogenic bladder is the restriction related to the compatibility of InterStim with magnetic resonance imaging.

The actual InterStim model II 3058 is authorized for head only 1.5 Tesla MRI; therefore, current recommendations suggest the need for SNM explants in patients requiring MRI for a non-cranial indication. This is indicated to be the reason for removal in more than 23% of InterStim removal surgeries, with 10% of the patients deciding to replace their devices at a later date (59).

Nevertheless, multiple studies have indicated that the use of MRI with the current SNM device is safe, including an exvivo phantom model which demonstrated that the intact system didn't undergo significant heating (60). Another study, by the same institution had 11 patients receive lumbar MRI with an intact system and recorded no adverse events (61). This limitation has motivated the research and development of new MRI-conditional equipment in the industry. Currently, the Axonics SNM system™,and a new InterStim model II with MRI-safe leads (SureScan leads)is available in many countries.

A number of studies have demonstrated the success of the Axonics system. The RELAX-OAB trial, a multicenter prospective clinical trial of 51 OAB patients treated with the Axonics SNM system™ noted that 71% were responders at 4 weeks (62). Of those patients, 94% continued to respond within 1 year, with an average reduction of 2.5 to 0.4 daily episodes of incontinence, and a decrease in urinary frequency episodes from 14 to 8 times per day.

In addition, total continence was achieved in 23%. In addition the ARTISAN trial (discussed earlier) noted significant improvements as well. These short-term results are comparable with results using the InterStim™ SNM device. Further differences of the Axonics SNM system™ are a decreased size of the implantable pulse generator, MRI compatability and a rechargeable device.

The new InterStim model II with SureScan MRI-safe leads which is also MRI compatible, rechargeable and has a very small IPG is commercially available in some countries.

### Spinal cord injury:

Sievert et al. published a case series of 4 patients subjected to SNM therapy shortly after a complete spinal cord injury who evolved to having no incontinence or detrusor overactivity (63).

This potential application of SNM could change the current practice in neurogenic bladder patients.

## TECHNIQUE POINTERS

### Success

The ICS guidelines state that “Clinicians should strive to achieve proper motor and/or sensory responses on all 4 contacts at stimulus amplitudes of <2 volts” (1).

A well-placed SNM lead should provide enough therapeutic stimulation from numerous electrodes and allow reprogramming a damaged electrode, should it be necessary (64).

A recent study, demonstrated that patients with greater electrode response scores had a lower probability of undergoing a device revision, and a lower likelihood of revision was evident with better toe motor response scores, though not bellows scores (65).

This may be explained by the fact that a great toe response depends essentially on a single muscle, while the pelvic bellows response may be generated from many muscles. Thus a toe response emulates stimulation of a minor and more therapeutically significant target nerve, whereas pelvic bellows appear stimulating any part of a broader target, perhaps various nerves (65).

Therefore, more precision should be taken when placing the lead to achieve a toe response, making this a better predictor of lead placement and a consecutive lower possibility of requiring a lead revision.

In 2011, a study by Peter et al. showed no improvement in therapeutic results nor diminution of revision rates with intraoperative sensory testing at the time of implantation (66), but assessing the motor response could be convenient for optimizing lead placement.

Correct lead placement is essential. The authors of a manuscript that shows the evolution of SNM in urologic practice, emphasize that the leads should be placed superomedially, in the S3 foramina with outward lateral curvatures as these curves follow the anatomical course of the sacral nerve, and accomplishes responses during intraoperative testing at low amplitudes (< 2 volts) (67). This inherently achieves greater total electrode response score and a higher toe score which are both correlated to a decreased risk for device revision (65).

This decreased risk for revision probably results from an enhanced ability to “reprogram” around a defective or misplaced electrode to another one that gives therapeutic benefit, making it possible for the device to remain intact and avoiding a surgical revision (64).

Besides electrode movement, things like nervous system accommodation could also explain variations in efficacy of the original programmed electrode. In such occurrences, the substitute electrode should be adequately close to the target nerve to employ its effect, which could point out why having a larger number of electrode responses is protective from device revision (65).

Furthermore, as any SNM device will need battery replacement revision, optimizing lead positioning may aid by delaying the requirement of future revisions by not only enabling easier reprogramming, but also increasing its battery life. The closer the lead to the target nerve, the lower the amplitude needed to stimulate it. This also prolongs battery life and conserves device life. So, factors that optimize lead placement must be taken into consideration (65).

Research regarding SNM outcome optimization is greatly needed because as of today the only validated tool that measures correct lead placement is motor response, which is an indirect way to measure lead placement.

## FUTURE OF SACRAL NEUROMODULATION?

The current implantable device (InterStim, Medtronic) has several weaknesses that need to be addressed. For instance, it is an expensive device, which explains the use of a staged-implant approach (1). Other noted deficiencies include battery life, lead fracture, MRI compatibility and clinician-dependent programming, which need to be improved, as does its long-term effect on bladder and bowel physiology (1). As noted above new devices that address some of these issues have recently become available.

Surgical technique has remained unchanged since the introduction of the tined-electrode, which disposed of the necessity to suture directly to the fascia. Nevertheless, there is an ongoing debate in regard to how accurately the lead must be positioned. There is evidence that suggests that only one active electrode is needed for a proper clinical response (68), but most studies encourage the use of 4-electrodes targeted at low voltages (69). CT supervision could be helpful in those with complex anatomical findings (69), but intraoperative EMG may also be of help in specific cases (70). Even so, more outcome-based research is needed to define the best method for introducing the lead.

Moreover, other than the 4-program settings and patient selection based on perceived symptom amelioration, specific programming recommendations are lacking.

New approaches may incorporate the patient's choice for a program, based on a real time bladder diary. Adding to this, smartphone apps are already available for patients to track their symptoms and could be used in device programming (71).

Economic modeling proposes that SNM becomes cost-effective in relation to intradetrusor botulinum toxin injections and to oral medical therapy for idiopathic OAB after 5 and 10 years respectively (72, 73). Nevertheless, there is little evidence on SNM cost-effectiveness relative to other therapies for fecal incontinence and urinary retention.
